# Age-related increases in right hemisphere support for prosodic processing in children

**DOI:** 10.1038/s41598-023-43027-8

**Published:** 2023-09-22

**Authors:** Kristen E. Li, Andrew Dimitrijevic, Karen A. Gordon, Elizabeth W. Pang, Hansel M. Greiner, Darren S. Kadis

**Affiliations:** 1 Department of Physiology, University of Toronto, Toronto, ON Canada; 2 Neurosciences and Mental Health, Hospital for Sick Children, 686 Bay Street, Toronto, ON M5G 0A4 Canada; 3 Department of Otolaryngology, Sunnybrook Health Sciences Centre, Toronto, ON Canada; 4 Department of Otolaryngology, University of Toronto, Toronto, ON Canada; 5 Division of Neurology, Hospital for Sick Children, Toronto, ON Canada; 6 Division of Neurology, Cincinnati Children′s Hospital Medical Center, Cincinnati, OH USA; 7 Department of Pediatrics, College of Medicine, University of Cincinnati, Cincinnati, OH USA

**Keywords:** Cortex, Language

## Abstract

Language comprehension is a complex process involving an extensive brain network. Brain regions responsible for prosodic processing have been studied in adults; however, much less is known about the neural bases of prosodic processing in children. Using magnetoencephalography (MEG), we mapped regions supporting speech envelope tracking (a marker of prosodic processing) in 80 typically developing children, ages 4–18 years, completing a stories listening paradigm. Neuromagnetic signals coherent with the speech envelope were localized using dynamic imaging of coherent sources (DICS). Across the group, we observed coherence in bilateral perisylvian cortex. We observed age-related increases in coherence to the speech envelope in the right superior temporal gyrus (*r* = 0.31, *df* = 78, *p* = 0.0047) and primary auditory cortex (*r* = 0.27, *df* = 78, *p* = 0.016); age-related decreases in coherence to the speech envelope were observed in the left superior temporal gyrus (*r* = − 0.25, *df* = 78, *p* = 0.026). This pattern may indicate a refinement of the networks responsible for prosodic processing during development, where language areas in the right hemisphere become increasingly specialized for prosodic processing. Altogether, these results reveal a distinct neurodevelopmental trajectory for the processing of prosodic cues, highlighting the presence of supportive language functions in the right hemisphere. Findings from this dataset of typically developing children may serve as a potential reference timeline for assessing children with neurodevelopmental hearing and speech disorders.

## Introduction

The seminal work of Broca^1^ and Wernicke^2^ led to a left hemisphere model for language, but recent studies have demonstrated a role for the right hemisphere, primarily in the processing of prosody^3^. Prosody refers to the stress, rhythm, and pitch changes in speech, which can convey information that is both emotional and linguistic in nature^4^. Emotional prosody refers to cues about the speaker’s affect or attitude^5^. Linguistic prosody refers to cues about the language structure such as lexical stress contrasts (e.g., *per*mit vs per*mit*) and word boundaries (e.g., *hotdog* vs *hot dog*)^6,7^. Lesion studies in adults have suggested that both types of prosody are processed by the right hemisphere, though some have argued that the left hemisphere is still involved, at least in the processing of linguistic prosody^8^.

In children, the neural bases of prosodic processing have not been well studied. Right hemisphere injuries are known to affect prosodic processing in childhood^9–11^, though neuroimaging studies suggest bilateral representation^12,13^. In electroencephalography (EEG) and magnetoencephalography (MEG) studies, the speech envelope is often extracted for analyses because of its ability to capture the rhythm and stress patterns of speech^14^. The speech envelope does not contain pitch information, and therefore reflects primarily linguistic rather than emotional prosody, but has still been widely used to study prosodic processing in children. For example, an MEG study computed coherence between the speech envelope and low frequency brain rhythms, related to prosody, and found high coherence in the bilateral auditory and temporal regions^15^. Similarly, an EEG study demonstrated impaired processing of low frequency speech envelopes in children with dyslexia^16^. However, these studies employed laboratory stimuli or paradigms, such as isolated sentences or word pairs.

Recently, studies of prosodic processing in children have begun using natural continuous speech. For example, 9-month-old infants were shown to be capable of tracking prosodic stress in response to continuous infant-directed speech^17^. Another study demonstrated speech envelope coherence in children aged 7–8 years during a stories listening task was related to reading level^18^. Unfortunately, many of these studies involve participants of limited age ranges and fail to describe potential changes in neural support for prosodic processing across development.

Here, we used MEG to map brain regions coherent with the speech envelope during a stories listening paradigm in a large cohort of typically developing children. Based on the right hemispheric specialization of prosodic processing in adults, we hypothesize that neural coherence to the speech envelope will become increasingly right lateralized with age.

## Methods

### Participants

Eighty children (42 female; ages 4.03–18.99 years, mean = 11.58) participated in this study. All were typically developing native English speakers with no prior history of neurological or psychological deficit and no MEG or MRI contraindications. Informed written consent was obtained from all participants and/or their legal guardians. Compensation was provided for participation and travel costs. The study was approved by the Institutional Review Board at Cincinnati Children’s Hospital Medical Center (OH, USA; data collection site), and the Research Ethics Board at the Hospital for Sick Children (Toronto, ON, Canada; analysis site). All methods and analyses were performed in compliance with the relevant guidelines and regulations and in accordance with the Declaration of Helsinki.

### Behavioural measures

Participants were drawn from two studies employing different language assessments as part of their cognitive batteries. Thirty children completed the Peabody Picture Vocabulary Test (PPVT-4; Dunn and Dunn^19^) and the Expressive Vocabulary Test (EVT-2; Williams^20^), assaying receptive and expressive vocabulary, respectively. We compute the arithmetic mean for PPVT-4 and EVT-2, to estimate overall language ability. The remaining 50 participants completed the Clinical Evaluation of Language Fundamentals (CELF-4^21^); we report on the Core Language Score, here. All participants (n = 80) completed the Weschler Nonverbal Scale of Ability (WNV; Wechsler and Naglieri^22^), and the Edinburgh Handedness Inventory (EHI; Oldfield^23^).

### MEG acquisition

MEG data were recorded using a 275-channel whole-head CTF system (CTF MEG Neuro Innovations, Inc., Coquitlam, BC, Canada) at 1200 Hz sampling. Participants were awake, studied in supine position with localization coils placed over the nasion and pre-auricular points for continuous monitoring of head position.

Participants completed a passive stories listening paradigm that has been extensively used by our group (e.g., Barnes-Davis et al.^24–26^). Participants listened to five stories narrated with a neutral affect by a female native English speaker. Stimuli were delivered through bilateral ear inserts connected to a distal transducer and tubing system (Etymotic Research, IL, USA). Each story was presented one sentence at a time in trials that lasted 2–3 s. After each story trial, participants listened to a noise trial consisting of a speech-shaped noise with identical duration, spectral content, and amplitude envelope.

### MRI acquisition

Prior to MR imaging, radiopaque fiducial markers were placed over the nasion and preauricular points to facilitate offline co-registration with MEG data. Whole-brain 3D T1-weighted images were acquired at 1 mm isotropic on a 3.0 T Philips Achieva or Ingenia Elition scanner (flip angle = 8°, TE = 3.5 ms, TR = 7.9 ms).

### Data analysis

#### Data preprocessing

MEG data were preprocessed using FieldTrip^27^ routines running in MATLAB R2020b (MathWorks Inc., Natick, MA, USA). The continuous MEG data were bandpass filtered from 0.1 to 100 Hz and power line noise (60 Hz) was attenuated using a 1 Hz-width notch filter. The data were then downsampled to 256 Hz and decomposed into 30 components using InfoMax Independent Component Analysis (ICA)^28^. The number of components for the ICA decomposition was chosen based on previous research^29,30^. Eyeblink and cardiac artifacts were visually identified based on their topographic map and time series, then subsequently rejected (mean number of rejected components = 1.94). Following artifact scrubbing, the data were segmented into 48 epochs of interest, each 3700 ms in duration.

#### Speech envelope extraction

The speech envelope was calculated as the absolute Hilbert transform of the speech signal, low-pass filtered at 10 Hz (forward and reverse) using a third-order Butterworth filter, and downsampled to 256 Hz to match the scrubbed MEG data.

#### Head and source modelling

For each participant, a realistic single-shell head model was constructed based on individual T1-weighted MRIs^31^. A whole-brain source-model was constructed based on the centroids of a ‘100-unit per hemisphere’ parcellation scheme^32^; the approach yielded 184 locations, across the cerebrum and cerebelli. For each participant, individual source models were generated by warping template positions to individual space.

#### Source reconstruction and coherence analysis

For each participant, trials were zero-padded to 4 s, filtered from 0.5 to 12 Hz to isolate prosodic and phonemic rhythms, and converted to the frequency domain using a multi-taper fast Fourier transformation with ± 2 Hz smoothing. A cross-spectral density matrix was computed, and stories and noise trials were concatenated for covariance estimation and subsequent spatial filter construction; however, only the stories data were assessed for coherence to the speech envelope using Dynamic Imaging of Coherent Sources (DICS) beamforming^33^. Individual template positions were then relabelled using the initial 3D template positions defined in MNI space.

## Results

### Behavioural results

Participants scored average to above average for language assessments (mean for combined PPVT-4 EVT-2 scores = 117.5, SD = 14.5; mean CELF-4 Core Language Score = 106, SD = 13.5). Nonverbal performance was within the average range (mean WNV Total = 107.1, SD = 13.1). The EHI score is computed as a conventional laterality index; we divide the range into 3 equal categories for handedness classification. Across the group, 90.0% of participants were right-handed, 6.25% left-handed, and 3.75% ambidextrous.

### Regions supporting speech envelope tracking in childhood

**Figure 1:**
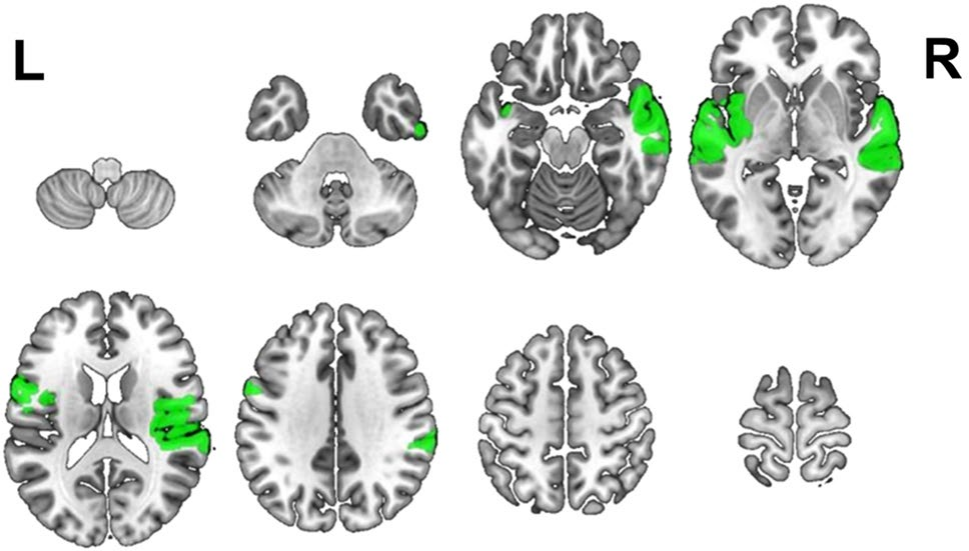
Regions supporting speech envelope tracking during a stories listening task. Ten perisylvian regions (parcels) showed significant coherence to the speech envelope (> 2 standard deviations above the mean). Each significant parcel is coloured in green and displayed on select axial slices. Corresponding statistical and anatomical information can be found in Table 1.

To identify regions coherent with the speech envelope across the group, Z-scores were computed for speech envelope coherence, for each node. A total of 10 regions had coherence values 2 standard deviations greater than the mean (Fig. 1, Table 1). Coherence to the speech envelope was localized in the bilateral frontal and temporal lobes, the right supramarginal gyrus, and the left insula. These 10 regions were used in subsequent correlation analyses, to assess developmental changes in speech envelope tracking.

**Table 1:** Statistical and anatomical information for regions supporting speech envelope tracking during a stories listening task.

Z-score	MNI coordinates			Anatomical region
	x	y	z	
5.80	59	− 28	− 2	Right superior temporal gyrus
3.87	− 57	− 9	− 1	Left superior temporal gyrus
3.54	59	− 31	22	Right supramarginal gyrus
2.90	− 58	− 29	3	Left superior temporal gyrus
2.78	41	− 22	14	Right primary motor
2.73	59	− 6	5	Right primary auditory
2.71	− 40	− 5	0	Left insula
2.70	61	− 18	− 24	Right medial temporal gyrus
2.41	55	0	− 15	Right superior temporal gyrus
2.23	− 56	4	18	Left pars opercularis (Broca's Area)

Z-scores were computed from the mean coherence values for each of the 184 regions of interest. Ten perisylvian regions (parcels) showed significant coherence to the speech envelope (> 2 standard deviations above the mean) and are shown in Fig. 1. *MNI* Montreal Neurological Institute.

### Age-related changes in speech envelope tracking

**Figure 2:**
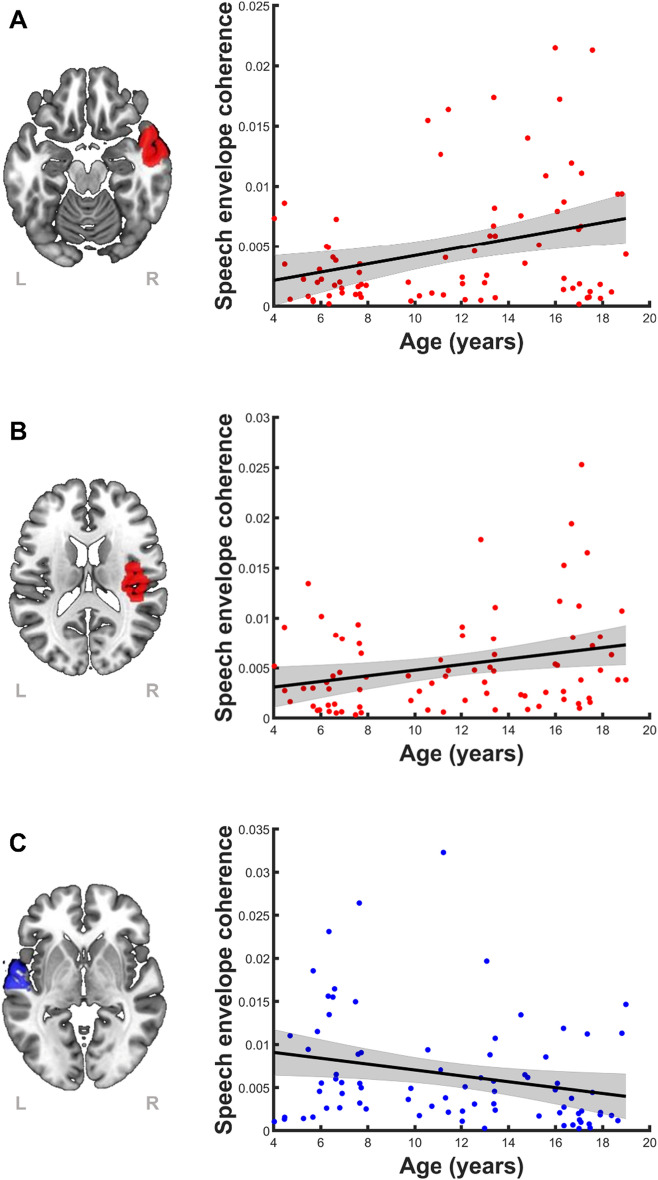
Age-related changes in speech envelope tracking (left) and corresponding scatterplot of coherence to the speech envelope as a function of age across the entire sample (*n* = 80) for each region of interest (right). Significant results passed an FDR of *q* = 0.1. (**A**) Age-related increases in coherence to the speech envelope were observed in the right superior temporal gyrus (*r* = 0.31, *p* = 0.0047), and (**B**) the right primary auditory cortex (*r* = 0.27, *p* = 0.016). (**C**) Significant age-related decreases in coherence to the speech envelope were observed in the left superior temporal gyrus (*r* = − 0.25, *p* = 0.026).

Pearson correlations for age versus speech envelope coherence was computed for each of the 10 regions showing significant speech tracking (see, Fig. 1). To control the Type I error rate, we applied an FDR (*q* = 0.01) across analyses. We observed significant age-related increases in coherence to the speech envelope in the right superior temporal gyrus (*r* = 0.31, *df* = 78, *p* = 0.0047) and the right primary auditory cortex (*r* = 0.27, *df* = 78, *p* = 0.016), and significant age-related decreases in the left superior temporal gyrus (*r* = − 0.25, *df* = 78, *p* = 0.026) (Fig. 2).

For each of the 3 significant regions of interests, partial correlations were computed between speech envelope coherence and age, sex, and handedness; the partial correlations permit characterization of bivariate relationships, while controlling for the other variables. In all cases, only age was significantly correlated to speech envelope coherence (Table 2).

**Table 2:** Partial correlations between speech envelope coherence and age, sex, and handedness for the three ROIs with significant age-related changes in speech envelope coherence shown in Fig. 2.

	Age		Sex		Handedness	
	*r*	*p*	*r*	*p*	*r*	*p*
R. primary auditory	0.28	0.013	0.12	0.27	0.0041	0.97
R. superior temporal	0.3	0.0077	− 0.058	0.61	0.062	0.59
L. superior temporal	− 0.23	0.041	0.085	0.46	− 0.06	0.6

*R*. right, *L*. left.

## Discussion

Here, we characterize maturation of the language network supporting the processing of prosodic cues. We assessed speech envelope tracking in children performing a stories listening task in MEG, and found that neuromagnetic signals coherent to the speech envelope localized to the bilateral perisylvian cortex. With age, coherence increased in the right temporal lobe and decreased in the left superior temporal gyrus. Findings suggest a distinct neurodevelopmental trajectory for the processing of prosodic cues, where areas in the right hemisphere become increasingly specialized.

Across the group, coherence to the speech envelope was localized to bilateral perisylvian cortex, including the right primary auditory cortex, the right supramarginal gyrus, the bilateral superior temporal gyri, left insula, and the left frontal lobe. These regions are known for auditory processing and language comprehension. Our results are consistent with speech envelope tracking studies in adults^34–36^. The shared regions among adults and children suggest that maturation of speech envelope tracking involves functional reorganization of pre-existing brain regions rather than anatomical reorganization of the network over time. Our coherence values are also consistent with previous studies of neural coherence to the speech envelope in adults^34,35^.

With age, coherence to the speech envelope increased in right temporal lobe and decreased in one region of the left superior temporal gyrus. These age-related differences are consistent with a previous MEG study that compared typically developing children to healthy adults^37^. The adult group had higher coherence to the speech envelope than children in a temporal region of interest that included the superior temporal gyrus. Thus, results suggest that young children use a mix of left and right language areas to track the speech envelope, with progressive support by right perisylvian regions with age, as the left perisylvian regions become less tuned to prosodic information related to the speech envelope. This pattern may provide insight into the brain differences underlying the improvement in prosodic processing during development, where the reallocation of neural resources creates a more efficient process.

We note that our study is limited by the omission of discrete pitch reference signals. Pitch is particularly important for conveying emotional information (i.e., emotional prosody)^38^. Others have documented age-related increases in right hemispheric activity in response to pure tones^39^. Thus, the processing of pitch may develop in a similar manner, as both pitch tracking and pure tones tracking involve attending to the fundamental frequency of a sound wave. Our stories were narrated by a single speaker who was instructed to speak with a neutral affect. As such, the stimuli have reduced emotional prosody, compared to entirely naturalistic stimuli. Future research involving speech envelope tracking in children should also include speech and noise contrasts, variations in speech-in-noise ratios, or speech containing irony, sarcasm, or other dialogues dependent on prosodic cues. Finally, future large-scale studies may have better sensitivity to potential nonlinearities in age-related effects not observed with the current cohort (see Sharma et al. 2021^40^, for example). Altogether, our results reveal a strong neurodevelopmental trajectory for the processing of prosodic cues and highlight the presence of supportive language functions in the right hemisphere. The results may potentially serve as a reference, where the maturational stage of children with language impairments can be compared to that of their typically developing peers. Our study emphasizes the importance of considering suprasegmental features in language development research. The precise timing of the language network’s reorganization, including its supportive functions, may be crucial for predicting language impairments and informing therapeutic practices.

## Data Availability

The data analyzed in the current study are not publicly available. Code used for analyses were developed from open-source toolboxes; scripts will be made available from the corresponding author on reasonable request.
